# Prevalence, Mortality, and Access to Care for Chronic Kidney Disease in Medicaid-Enrolled Adults With Sickle Cell Disease in California: Retrospective Cohort Study

**DOI:** 10.2196/57290

**Published:** 2024-07-15

**Authors:** Jhaqueline Valle, Jeffrey D Lebensburger, Pranav S Garimella, Srila Gopal

**Affiliations:** 1 Tracking California Public Health Institute Oakland, CA United States; 2 Division of Pediatric Hematology-Oncology Department of Pediatrics University of Alabama at Birmingham Birmingham, AL United States; 3 Division of Nephrology-Hypertension Department of Medicine University of California San Diego San Diego, CA United States; 4 Division of Hematology/Oncology Department of Medicine University of California San Diego La Jolla, CA United States

**Keywords:** sickle cell disease, chronic kidney disease, prevalence, mortality, access to care, Medicaid, California, United States, retrospective, cohort study, investigate, emergency department, hospitalization, specialized care, adult, adults, hematologist, hematologists, nephrologist, nephrologists, t-test

## Abstract

**Background:**

Chronic kidney disease (CKD) is a significant complication in patients with sickle cell disease (SCD), leading to increased mortality.

**Objective:**

This study aims to investigate the burden of CKD in Medicaid-enrolled adults with SCD in California, examine differences in disease burden between male and female individuals, and assess mortality rates and access to specialized care.

**Methods:**

This retrospective cohort study used the California Sickle Cell Data Collection program to identify and monitor individuals with SCD. Medicaid claims, vital records, emergency department, and hospitalization data from 2011 to 2020 were analyzed. CKD prevalence was assessed based on *ICD* (*International Classification of Diseases*) codes, and mortality rates were calculated. Access to specialized care was examined through outpatient encounter rates with hematologists and nephrologists.

**Results:**

Among the 2345 adults with SCD, 24.4% (n=572) met the case definition for CKD. The SCD-CKD group was older at the beginning of this study (average age 44, SD 14 vs 34, SD 12.6 years) than the group without CKD. CKD prevalence increased with age, revealing significant disparities by sex. While the youngest (18-29 years) and oldest (>65 years) groups showed similar CKD prevalences between sexes (female: 12/111, 10.8% and male: 12/101, 11.9%; female: 74/147, 50.3% and male: 34/66, 51.5%, respectively), male individuals in the aged 30-59 years bracket exhibited significantly higher rates than female individuals (30-39 years: 49/294, 16.7%, *P*=.01; 40-49 years: 52/182, 28.6%, *P*=.02; and 50-59 years: 76/157,48.4%, *P*<.001). During this study, of the 2345 adults, 435 (18.5%) deaths occurred, predominantly within the SCD-CKD cohort (226/435, 39.5%). The median age at death was 53 (IQR 61-44) years for the SCD-CKD group compared to 43 (IQR 33-56) years for the SCD group, with male individuals in the SCD-CKD group showing significantly higher mortality rates (111/242, 45.9%; *P*=.009) than female individuals (115/330, 34.9%). Access to specialist care was notably limited: approximately half (281/572, 49.1%) of the SCD-CKD cohort had no hematologist visits, and 61.9% (354/572) did not see a nephrologist during this study’s period.

**Conclusions:**

This study provides robust estimates of CKD prevalence and mortality among Medicaid-enrolled adults with SCD in California. The findings highlight the need for improved access to specialized care for this population and increased awareness of the high mortality risk and progression associated with CKD.

## Introduction

Chronic kidney disease (CKD) presents a significant complication in sickle cell disease (SCD), marked by its high prevalence and a notable increase in mortality. Nearly 70% of adults with SCD develop albuminuria [1], and roughly 20% progress to overt CKD, characterized by a glomerular filtration rate (GFR) of less than 60 ml/min/1.73 m^2^ [2]. Each year, over 100 patients with SCD advance to end-stage renal disease (ESRD) [3]. The mortality risk in patients with SCD-related ESRD is 3-fold higher compared to those without CKD [4], particularly with proteinuria. Key predictors of mortality in SCD are a reduced GFR and rapidly deteriorating kidney function [5-7], contributing to 16% to 18% of overall mortality in SCD [8]. Increasing age is a risk factor for CKD [9], and male individuals are more likely to have a faster and more rapid kidney function decline [10]. As life expectancy in patients with SCD increases [11], the burden of SCD-associated CKD is expected to rise.

While the impact of CKD on SCD outcomes is well recognized, contemporary data on its prevalence and burden, especially in the aging population with SCD, are limited. This is particularly true for patients outside of specialized sickle cell clinics. Most current prevalence estimates are derived from pediatric sickle cell centers [2] or older patient cohorts [12]. However, the situation for adults with SCD is starkly different. Comprehensive care for adults is notably lacking [13], and most do not have access to specialized hematological care [14]. Thus, patients who are followed at established sickle cell centers may not represent the entire population of patients with SCD, and CKD in the population with SCD may be underrecognized. Furthermore, the majority of patients with SCD, often reliant on Medicaid, face significant barriers to accessing specialized care [15]. Access to timely and early nephrology care can impact the treatment of CKD and subsequent complications. Thus, more robust and contemporary population-wide approaches may help us in improving our understanding of the burden and prevalence of CKD in adults with SCD.

To address the current limitations in the field, our study aims to investigate the burden of CKD among Medicaid-enrolled adults with SCD in California using the Sickle Cell Data Collection (SCDC) program’s large administrative database over a 10-year time frame (2011-2020). The SCDC program offers a comprehensive view as a state-based, population-wide public health surveillance system for SCD [16]. Our report estimates the prevalence of CKD in the population with SCD by age groups, providing insights into mortality rates and access to specialized care, both hematology and nephrology, for adults with and those without CKD.

## Methods

### Study Design

This is a retrospective cohort study of Medicaid-enrolled people with SCD in California. All study data were obtained from the California SCDC program. The SCDC program leverages a variety of data sources to identify and longitudinally monitor individuals with SCD within the state. These data sources include newborn screening, nonfederal hospital discharge records, emergency room visits, ambulatory surgery encounters, vital records death files, Medicaid claims, and enrollment data, as well as clinical case reports from SCD care centers within the state. The data are linked and deduplicated across various sources and multiple years. Detailed information about the SCDC program and case definitions have been previously published [16-19].

### Ethical Considerations

The SCDC program and this study were reviewed and approved by the California Committee for the Protection of Human Subjects (protocol 15-10-2249) and the Public Health Institute Institutional Review Board (protocol I16-001). California SCDC received a waiver of consent.

### Data Sources

Data sources used in this analysis include Medicaid claims, hospital discharge data, and vital records data from 2011 to 2020. The source data included all outpatient provider claims, inpatient hospital, emergency department encounters, and all-cause mortality.

### Study Population

Individuals aged 18 years and older, who met the case definition for SCD, were enrolled in Medicaid in 2011, and who maintained their Medicaid coverage for at least 75% (75/100) of their time in this study were included. For example, if someone’s enrollment spanned 6 years, they were required to be enrolled for at least 4.5 years cumulatively, regardless of whether the enrollment was continuous.

### Study Measures

The demographic characteristics of individuals are reported by the total study population. Age was calculated on the first date of this study, January 1, 2011. SCD subtype was recorded from State Newborn Screening records or clinical case reports from SCD centers. Subtypes were categorized as hemoglobin (Hb)–SS or Hb-Sβ^0^ thalassemia (sickle cell anemia), Hb-Sβ^+^ thalassemia, Hb-SC, other compound homozygous forms of SCD, or unknown if laboratory confirmation was not available within the SCDC database. An individual was considered dual eligible, meaning they were enrolled in both Medicare and Medicaid, if any Medicaid claims data indicated dual eligibility. For the prevalence estimation, age is calculated at the last date of this study, December 31, 2020, or date of death, whichever came first. Disposition codes within the emergency department and hospitalization data as well as linked vital death records were used to identify all-cause deaths occurring during this study’s period. The proportion of deaths was calculated as the number of people with CKD who died, regardless of cause, by age group divided by the total number of people who developed CKD by age group, calculated at age on the last date of this study, December 31, 2020, or date of death, whichever came first.

We ascertained individuals’ CKD status (none versus any, the latter encompassing stages 1 through 5, unspecified stage, and ESRD), stage of CKD is based on *ICD-9-CM* (*International Classification of Diseases, Ninth Revision, Clinical Modification*) and *ICD-10-CM* (*International Classification of Diseases, Tenth Revision, Clinical Modification*) diagnosis codes. Individuals met the case definition for CKD if, within 5 years, they had 3 or more outpatient or emergency department encounters with CKD-coded diagnoses (*ICD-9-CM*: 585, 5851-5856, or 5859; *ICD-10-CM*: N18, N181-N185, or N188-N189) recorded in any diagnostic position, or at least 1 hospitalization with such codes in any position [20]. We identified a cohort of 572 individuals who by the end of this study’s period met the case definition for CKD. Subsequently, we will reference this subset as the SCD-CKD cohort. The remaining that did not meet the case definition will be referred to as the SCD cohort.

Hematologist and nephrologist encounters were identified using the National Provider Identifier of the rendering provider, as listed in the Medicaid claims records [21]. Providers with any health care provider taxonomy code listed as hematologist (207RH0000X, 207RH0003X, or 2080P0207X) were categorized as a hematologist; nephrologists were categorized as such if they had any health care provider taxonomy code listed as a nephrologist (207RN0300X). To calculate an individual’s total number of encounters with a hematologist (both groups) or nephrologist (CKD only), Medicaid claims were deduplicated by stipulating that an individual could only be recorded as having 1 encounter with a specific provider per day. The outpatient encounter rate was calculated by dividing the total number of unique encounters by the total person-years attributed to each group. To calculate the proportion of individuals who had no visits with a nephrologist or hematologist, the entire study duration was examined, and those with no encounters with either specialist were identified.

### Statistical Analysis

Categorical variables were summarized using frequencies and percentages and compared for statistical significance using chi-square tests. Continuous variables were summarized by means and rates, and the Wilcoxon-Mann-Whitney *U* test was used to test for differences between SCD-CKD and the SCD groups. All analyses were performed using SAS (version 9.4; SAS Institute Inc).

## Results

### Characteristics of Study Cohorts

Table 1 presents the demographic characteristics of the 2345 individuals in this study’s cohort from 2011 to 2020.

**Table 1:** Demographic characteristics of individuals included in this study^a^.

Characteristics		Total (N=2345)	SCD^b^-CKD^c^ (n=572)	SCD (n=1773)
**Age group (years), n (%)**				
	18-29	916 (39.1)	105 (18.4)	811 (45.7)
	30-39	530 (22.6)	107 (18.7)	423 (23.9)
	40-49	453 (19.3)	148 (25.8)	305 (17.2)
	50-59	308 (13.1)	136 (23.8)	172 (9.7)
	60-64	65 (2.77)	32 (5.6)	33 (1.9)
	65+	73 (3.1)	44 (7.7)	29 (1.6)
Age^d^ (years), mean (SD)		36 (17.7)	44 (14)	34 (12.6)
**Sex, n (%)**				
	Female	1495 (63.8)	330 (57.7)	1165 (65.7)
	Male	850 (36.3)	242 (42.3)	608 (34.3)
**SCD subtype** **, n (%)**				
	SCA^e^ (Hb^f^-SS or Hb-Sβ^0^ Thal^g^)	332 (14.2)	96 (16.8)	236 (13)
	Hb-Sβ^+^ Thal or Hb-SC	144 (6.14)	28 (4.9)	116 (6.5)
	Unknown	1869 (79.7)	448 (78.3)	1421 (80.2)
Dual eligible, n (%)		941 (40.1)	333 (58.2)	608 (34.3)

^a^Met case definition by the end of this study period.

^b^SCD: sickle cell disease.

^c^CKD: chronic kidney disease.

^d^Age at the start of this study from January 1, 2011.

^e^SCA: sickle cell anemia.

^f^Hb: hemoglobin.

^g^Thal: thalassemia.

At the start of this study’s period, the SCD-CKD group (n=572) were older in age, with nearly half (n=284, 49.7%) being in the aged 40-59 years categories (n=148, 25.8%, 40-49 years; n=136, 23.8%, 50-59 years). The mean age at the start of this study for the SCD-CKD cohort was 44 (SD 14) years. In contrast, the SCD cohort (n=1773) had an average age of 34 (SD 12.6) years, with 45.7% (n=811) of them between the ages of 18 and 29 years. In both the SCD-CKD and SCD groups, female individuals were more predominant at 57.7% (330/572) and 65.7% (1165/1773) respectively. Among the people with a known genotype across both groups, sickle cell anemia was more prevalent than Hb-Sβ^+^ thalassemia or Hb-SC. However, nearly 79.7% (1869/2345) had unknown genotypes. More than half (333/572, 58.2%) of the SCD-CKD cohort were considered dual eligible for Medicaid and Medicare.

### CKD Prevalence by Age Group and Sex

**Figure 1:**
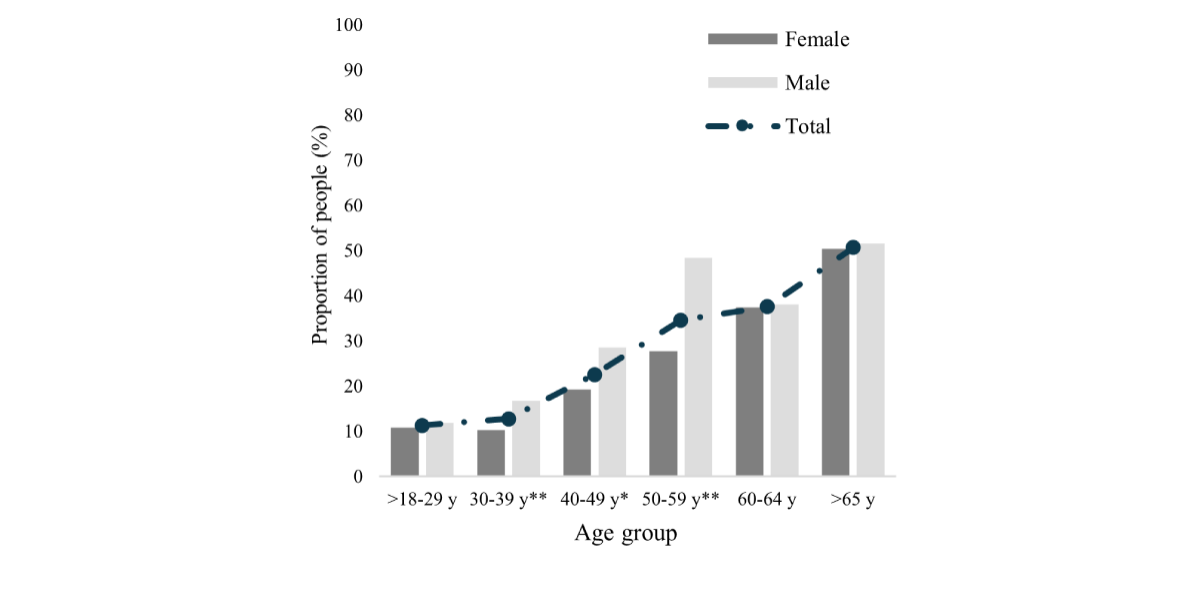
Sex-specific prevalence of chronic kidney disease among individuals with sickle cell disease by age group, 2011-2020. Fisher exact *χ*^2^ test: *P<.05; **P<.001.

The prevalence of CKD by age group and sex, as presented in Figure 1, shows a notable pattern of increasing prevalence with advancing age, alongside a distinct variation between female and male individuals. In the youngest age group (18-29 years) and oldest age groups (>65 years), the prevalence is similar between female and male individuals, at 10.8% (12/111) female and 11.9% (12/101) male and 50.3% (74/147) female and 51.5% (34/66) male, respectively. Male individuals have a significantly higher CKD prevalence in the 30 (*P*=.01), 40 (*P*=.02), and 50 (*P*<.001) years age groups. The highest prevalence is observed in the age group of 65 years and older, with 50.3% (74/147) in female individuals and 51.5% (34/66) in male individuals, resulting in a combined prevalence of 50.7% (108/213). These estimates are from the end of this study’s period.

### CKD and Mortality

**Figure 2:**
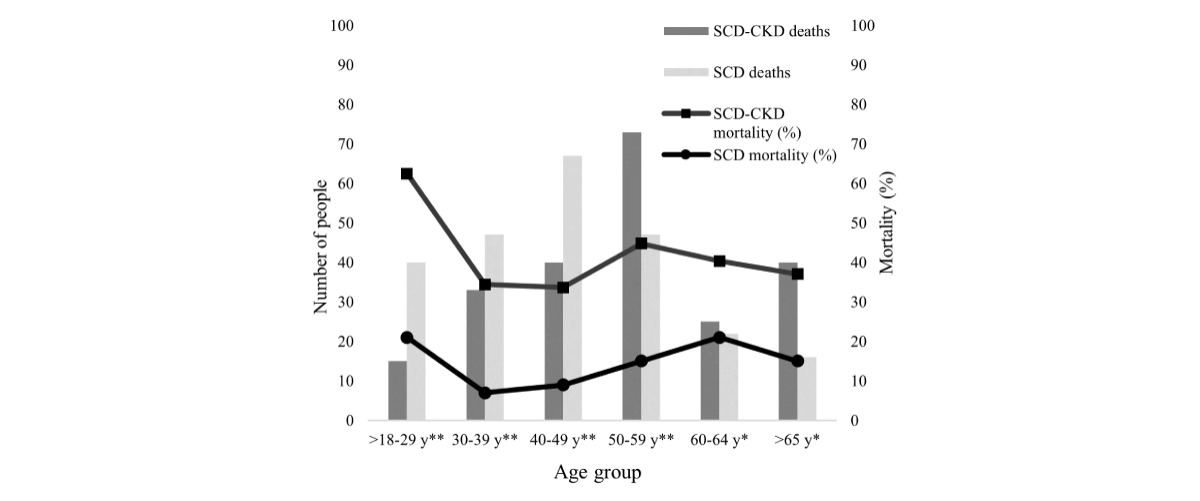
Mortality trends by age group in individuals with sickle cell disease, with and without chronic kidney disease, 2011-2020. Fisher exact *χ*^2^ test: *P<.05; **P<.001. CKD: chronic kidney disease; SCD: sickle cell disease.

Of the 2345 adults, there were 435 (18.5%) deaths that occurred during this study’s period, with the majority of deaths occurring in the SCD-CKD cohort (226/572, 39.5% of the SCD-CKD group). The median age at death was 53 (IQR 61-44) years for the SCD-CKD cohort and 43 (IQR 33-56) years for the SCD cohort. When compared to the SCD cohort, the SCD-CKD cohort had higher death rates for all age groups (Table 2). The lowest total number of deaths was identified in the youngest age group with SCD-CKD (>18-29 years); however, we identified a 63% mortality rate among individuals with SCD-CKD in this youngest age group. The remaining age groups also had high rates of mortality that ranged from 34% (33/96; 30-39 years) to 45% (73/163) among the 50-59 years age group (Figure 2). Male individuals with SCD-CKD had a significantly higher rate of mortality (45.9%;111/242, *P*=.009) compared to female individuals (34.9%, 115/330) with SCD-CKD; this trend, although not statistically significant, persisted across most age groups (Table 2).

**Table 2:** All-cause mortality rates by age group, sex, and chronic kidney disease status among individuals with sickle cell disease, 2011-2020.

Age group	SCD^a^-CKD^b^ (n=226/572)				SCD (n=209/1,773)		
	Female, n (%)	Male, n (%)	P value^c^	Female, n (%)		Male, n (%)	P value^c^
Total	115/330 (34.9)	111/242 (45.9)	.009	124/1165 (10.6)		85/608 (13.9)	.04
>18-29 years	CS^d^	CS	CS	17/99 (17.1)		23/89 (25.8)	.16
30-39 years	14/47 (29.8)	19/49 (38.8)	.40	24/414 (5.8)		23/245 (9.4)	.09
40-49 years	21/67 (31.3)	19/52 (36.5)	.56	21/280 (7.5)		16/130 (12.3)	.14
50-59 years	36/87 (41.3)	37/76 (48.7)	.43	33/227 (14.5)		14/81 (17.3)	.59
60-64 years	CS	CS	CS	CS		CS	CS
>65 years	25/74 (33.8)	15/34 (44.1)	.39	CS		CS	CS

^a^SCD: sickle cell disease.

^b^CKD: chronic kidney disease.

^c^Fisher exact chi-square test.

^d^CS: cell suppressed.

### Access to Care

**Figure 3:**
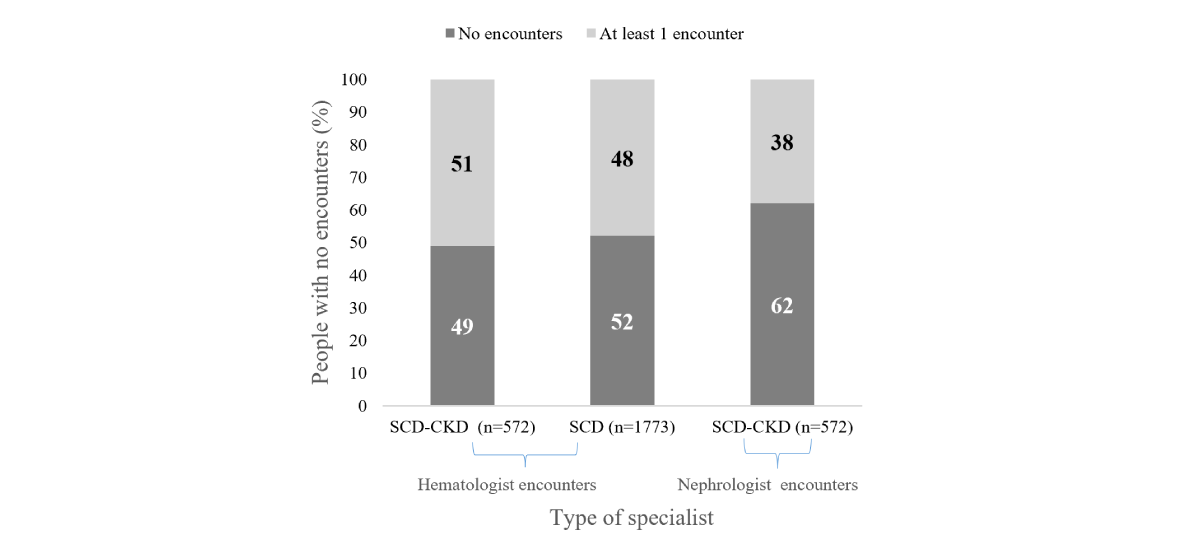
Proportion of individuals with sickle cell disease, with and without chronic kidney disease, who had 0 hematologist encounters for over 10 years, 2011-2020. CKD: chronic kidney disease; SCD: sickle cell disease.

Figure 3 and Table 3 show the rate of outpatient encounters with a hematologist or a nephrologist per person-year and the proportion of people with SCD-CKD who had zero visits with either specialist. Among those with SCD-CKD, visit rates with a hematologist were approximately 2 visits per person-year; however, almost half (281/572, 49.1%) of people had no encounters with a hematologist over the entire study period. Access to nephrology care was also limited; in the group with SCD-CKD, individuals had only approximately 1 visit to a nephrologist per person-year, and 61.9% (354/572) of people did not have any encounters with nephrologists throughout the entire study period.

**Table 3:** Specialist outpatient visit rates in individuals with sickle cell disease, with and those without chronic kidney disease, 2011-2020.

	SCD^a^-CKD^b^ (n=572)	SCD (n=1773)	*P* value
Rate of outpatient hematologist visits per person-year	2.1	1.5	<.001^c^
People with 0 visits with a hematologist^d^, n (%)	281 (49.1)	925 (52.1)	.21^e^
Rate of outpatient nephrologist visits per person-year	0.9	—^f^	—
People with 0 visits with a nephrologist^d^, n (%)	354 (61.9)	—	—

^a^SCD: sickle cell disease.

^b^CKD: chronic kidney disease.

^c^Mann-Whitney *U* test.

^d^During the entire study period.

^e^Fisher exact chi-square test.

^f^Not applicable.

In our analysis of the SCD-CKD group, comparing patients who are deceased to those who are alive at this study’s end, we found a significantly higher proportion of patients who are deceased had never consulted a hematologist (152/226, 67.2% vs 129/346, 37.3%; *P*<.001) or seen a nephrologist (154/226, 68.1% vs 200/346, 57.8%; *P*=.007), as shown in Table 4.

**Table 4:** Rate of outpatient visits with a specialist by mortality among SCD^a^-CKD^b^.

	Deceased (N=226)	Alive (N=346)	*P* value
Rate of outpatient hematologist visits per person-year	2.2	2.1	.52^c^
People with 0 visits with a hematologist^d^, n (%)	152 (67.2)	129 (37.3)	<.001^e^
Rate of outpatient nephrologist visits per person-year	1.3	0.8	<.001^c^
People with 0 visits with a nephrologist^d^, n (%)	154 (68.1)	200 (57.8)	.007^e^

^a^SCD: sickle cell disease.

^b^CKD: chronic kidney disease.

^c^Mann-Whitney *U* test.

^d^During the entire study period.

^e^Fisher exact chi-square test.

## Discussion

### Principal Findings

Our study estimates the prevalence and mortality associated with CKD in Californian adults with SCD using a large administrative database. Similar to other single and multi-institutional cohorts, we found a high prevalence of CKD (572/2345, 24.4%) among adults with SCD. Prevalence of CKD increased with age, with significant differences between male and female individuals. Mortality rates were higher in the SCD-CKD cohort. Access to specialized care was limited, with a significant proportion of individuals having no encounters with hematologists or nephrologists.

### Prevalence of CKD by Age Group and Sex

We identified 2345 adults (aged >18 years) during 10 years within the SCDC cohort. Of these, 572 fulfilled case definitions for CKD, with an estimated CKD prevalence of 24.4% (572/2345). Prior studies show a high prevalence of CKD in the population with SCD, and our study confirms these findings. Further, 1 observational study tracked 427 patients over 4 years, finding a baseline CKD prevalence of 21.4% in sickle cell anemia and 17.2% in Hb-Sβ^+^ thalassemia and Hb-SC SCD [2]. Studies from countries such as Nigeria and Ghana report even higher prevalence rates, around 38% and 39%, respectively [22,23]. Another study, a 25-year cohort study [24], identified the prevalence of renal failure at 4.2% in patients with sickle cell anemia (median age of onset was 23.1, range 13-57 years) and 2.4% in patients with Hb-SC SCD (median age of onset was 49.9, range 29-65 years). On additional follow-up, the prevalence increased to 12% [24]. The prevalence of CKD, calculated using the age at the end of this study or age at death, demonstrated an expected increase with advancing age. Our study continues to highlight the high prevalence of SCD-CKD in patients aged 18-29 years. By age >50 years, around 50% of patients will have developed SCD-CKD. Interestingly, our cohort predominantly comprised female individuals (1495/2345, 63.75%); this overrepresentation may be attributed to higher Medicaid coverage in female individuals, especially during their reproductive years [25]. Despite this female predominance, male individuals appear to have a higher prevalence of CKD compared to female individuals for each age category: a higher percentage of male individuals in our study had CKD (242/850, 28.5% vs 330/1495, 22.1%), deviating from the general population’s CKD distribution where female individuals slightly outnumber male individuals [26]. This finding further confirms sex differences in the prevalence of SCD-CKD at a population level [27]. This may be explained by the fact that male individuals with SCD have a higher rate of decline in GFR when compared to female individuals [28,29], leading to a greater burden of CKD.

While male individuals appear to have a higher prevalence of CKD for each age category, CKD prevalences for male and female individuals are the same in the aged 60-64 years group. This phenomenon may be partially explained by higher mortality rates for male individuals in the SCD-CKD cohort, and hence the comparable CKD rates in the oldest age groups may be because more male individuals with SCD-CKD are dying at a younger age. Taken together, these findings further emphasize the importance of the early CKD screening and intervention in SCD, especially in younger and male populations.

### CKD and Mortality

During this study’s period, 435 deaths occurred, translating to an overall mortality rate of 18.6% (435/2345). Notably, the mortality rate in the SCD-CKD cohort was high at 39.5% (226/572). Male individuals in the SCD-CKD cohort exhibited a higher death rate (45.9%, 111/242) compared to female individuals (34.9%, 115/330), deviating from previous studies that did not find a sex difference in mortality among patients with CKD and with SCD [1]. Notably, 50.9% (115/226) of the deaths in the SCD-CKD cohort occurred in individuals with ESRD, reinforcing the association of kidney disease with heightened mortality risk in SCD. The high mortality rate seen in our study is notably higher than the 18% found in the study by Platt et al [8]. In total, 1 key difference is the level of health care access between the 2 populations; the study by Platt et al [8] followed patients enrolled and regularly seen at established SCD centers, whereas nearly half or more of the patients included in this analysis never had an encounter with a hematologist (281/572, 49.1%) or nephrologist (354/572, 61.9%). In addition, the definition for renal failure was different in Platt et al [8], where they defined renal failure as a 20% increase in baseline creatinine concentration and a creatinine clearance rate below 100 mL/min [8], while we used *ICD* (*International Classification of Diseases*) codes to identify individuals with CKD. In our study, the median age at death was 10 years higher in the SCD-CKD cohort compared to the non-CKD SCD cohort, which was an unexpected finding given that CKD is a known risk factor for mortality in SCD [7]. This could be attributed to the older age distribution of the SCD-CKD cohort. This suggests that CKD may be more prevalent in the aging population with SCD, potentially characterizing it as a complication of the aging population with SCD and perhaps can be considered a disease of the “survivors.”

The significantly higher mortality rate in the SCD-CKD group, especially in younger age groups, is another important finding. The stark contrast in mortality rates between male and female individuals with SCD-CKD in the youngest age group indicates a need for focused research on gender-specific factors contributing to these outcomes. This elevated mortality risk associated with CKD in patients with SCD underscores the importance of the early detection and management of kidney disease.

### Access to Care

Severe lack of access to specialty services remains a critical issue for adults with SCD [14]. Notably, while the SCD-CKD group had more hematologist visits than the SCD group, nearly half had no specialist visits over the 10 years, highlighting a critical gap in specialty care access for adults with SCD. This lack of access is even more considerable given that 67.2% (152/226) of individuals with SCD and CKD who died had not consulted a hematologist, compared to 37.3% (129/346) among survivors. Furthermore, only 38.1% (n=218) of those with CKD had consulted a nephrologist, and likely only in the advanced stages of their disease. This is not a surprising observation, since it is likely that patients who had more access to care also had more access to preventative care, early detection of CKD, and likely disease-modifying strategies. Despite ESRD qualifying for Medicare, only 58.2% (333/572) were dual eligible, indicating that Medicaid coverage alone may not provide sufficient access to nephrology care [3]. This issue is not unique to the population with SCD and reflects broader systemic disparities in health care access, particularly for younger, predominantly Black and Hispanic individuals on Medicaid [3]. These findings emphasize the need for improved access to specialized care to better manage CKD and potentially reduce mortality in the population with SCD.

### Limitations

While there are several strengths to our study approach as outlined above, there are also several limitations. The major limitation of our study is that it is a retrospective administrative data-based study. The use of *ICD* codes to capture CKD diagnoses has been validated previously [20], however, we are likely underestimating the burden of CKD for multiple reasons. We did not include *ICD* codes for proteinuria since we felt this would be less reliable when relying on administrative data alone for data collection. While we included all stages of CKD, we likely underestimated CKD 1 and 2 where the GFR is >60 mL/min because not all of these may have been recognized or coded correctly. In assessing the limitations of our methodology for calculating the prevalence, it is important to consider the following aspects. First, individuals were categorized as either having CKD or not based on meeting the case definition at any point within a 10-year timeframe. This approach simplifies the classification process but may overlook cases of CKD who did not use health care or were not identified or documented with the relevant codes during the specified period. Additionally, because we used *ICD* codes within a set period of time, we could only identify when the first *ICD* CKD code appeared and not when a person was first diagnosed with CKD. Lastly, only Medicaid recipients were included in our study and about a third of patients with SCD may have commercial insurance or Medicare only and may not have been included in our analysis. In our assessment of care access, we focused exclusively on visits to hematologists, without distinguishing providers with specific training in SCD. Previous research indicates that adult patients with SCD in California face significant access barriers, and including primary care providers with SCD expertise—defined as having 20 or more patients with SCD—did not substantially improve access [14]. In addition, we could not ascertain whether individuals who disenrolled from Medi-Cal then enrolled in other health plans or became uninsured. While our data does not enable us to confirm whether individuals accessed specialty care during periods they were not enrolled in Medicaid, the literature suggests that accessing specialist services without insurance is highly unlikely [30]. Since this was an administrative database-based study, we had limited access to detailed clinical information, and could not adjust for covariates or perform robust statistical analysis, hence our results are presented descriptively.

### Conclusions

We used administrative data to estimate the prevalence of CKD in SCD among California state Medicaid recipients. Our prevalence estimates for CKD are higher than what has been reported previously. Those with SCD and concurrent CKD experienced significantly higher mortality rates compared to individuals with SCD who did not meet the case definition for CKD. In California, individuals with SCD face substantial barriers to accessing specialty care. Our findings highlight the critical need for this data to guide state health care stakeholders and government policy makers in addressing these access issues. As the SCDC program expands to include additional states, similar studies may inform our understanding of the true burden of CKD in the population with SCD, including its effects on mortality, morbidity, health care use, and quality of life.

## Abbreviations

CKD: chronic kidney disease
ESRD: end-stage renal disease
GFR: glomerular filtration rate
Hb: hemoglobin
ICD: International Classification of Diseases
*ICD-9-CM*: *International Classification of Diseases, Ninth Revision, Clinical Modification*
*ICD-10-CM*: *International Classification of Diseases, Tenth Revision, Clinical Modification*
SCD: sickle cell disease
SCDC: sickle cell data collection

## Data Availability

These data are sourced from third parties, and data use agreements that prohibit sharing even deidentified person-level data. Should readers wish to explore gaining access to the data, they may contact the original data stewards. Requests for minimal data set data can be directed to the corresponding author.
